# Hemispheric asymmetries and brain size in mammals

**DOI:** 10.1038/s42003-023-04894-z

**Published:** 2023-05-15

**Authors:** Sebastian Ocklenburg, Yasmin El Basbasse, Felix Ströckens, Anett Müller-Alcazar

**Affiliations:** 1 Department of Psychology, MSH Medical School Hamburg, Hamburg, Germany; 2 Institute for Cognitive and Affective Neuroscience, MSH Medical School Hamburg, Hamburg, Germany; 3 Biopsychology, Institute for Cognitive Neuroscience, Ruhr University Bochum, Bochum, Germany; 4 C. & O. Vogt Institute for Brain Research, University Hospital Düsseldorf, Heinrich-Heine University Düsseldorf, Düsseldorf, Germany

**Keywords:** Cognitive neuroscience, Evolutionary developmental biology

## Abstract

Hemispheric asymmetries differ considerably across species, but the neurophysiological base of this variation is unclear. It has been suggested that hemispheric asymmetries evolved to bypass interhemispheric conduction delay when performing time-critical tasks. This implies that large brains should be more asymmetric. We performed preregistered cross-species meta-regressions with brain mass and neuron number as predictors for limb preferences, a behavioral marker of hemispheric asymmetries, in mammals. Brain mass and neuron number showed positive associations with rightward limb preferences but negative associations with leftward limb preferences. No significant associations were found for ambilaterality. These results are only partly in line with the idea that conduction delay is the critical factor that drives the evolution of hemispheric asymmetries. They suggest that larger-brained species tend to shift towards more right-lateralized individuals. Therefore, the need for coordination of lateralized responses in social species needs to be considered in the context of the evolution of hemispheric asymmetries.

## Abstract (web summary)

Cross-species analysis of brain mass and neuron number suggest that larger-brained species tend to shift towards more right-lateralized individuals, which should be considered in the context of the evolution of hemispheric asymmetries moving forward.

## Introduction

The human brain shows a considerable number of functional left-right differences, such as the leftward bias for speech processing or the rightward bias for face processing^1–3^. Similarly, a wide range of non-human vertebrate and non-vertebrate species show such functional asymmetries across a wide range of cognitive systems^4–6^.

Interestingly, the extent and direction of hemispheric asymmetries differ considerably across species^7^. For example, humans show a clear population-level asymmetry for handedness, with roughly 90% of individuals being right-handed and 10% being left-handed^8^. Meta-analyses on paw preferences in rats and mice^9^ and cats and dogs^10^, however, did not reveal any population-level asymmetry in these species. Moreover, these species also showed a considerably higher number of individuals that did not have a clear preference for either side as compared to humans for which true ambidexterity is rare^11^. In common marmosets, ambilaterality is relatively rare and age-dependent, with younger animals showing ambilaterality more commonly in visuospatial reaching tasks^12^.

This led researchers to develop several theoretical models about why functional asymmetry would emerge as an organizational feature of nervous systems and which features of nervous systems influence the emergence and strength of hemispheric asymmetries. One influential hypothesis that has been suggested by Ringo in the 1990s^13^ is focused on the corpus callosum, the largest commissure connecting the two hemispheres in placental mammals^14^. Since the corpus callosum evolved in placental mammals, the more basal mammalian *Metatheria* do not possess a corpus callosum yet. Instead, the anterior commissure constitutes the major interhemispheric pathway^15^ and shows a connection pattern similar to the corpus callosum in placental mammals^16^. While other vertebrate species like birds, reptiles, amphibians, and fish do have similar commissural systems compared to *Metatherians*, they differ in extent and projection pattern, rendering the application of the Ringo hypothesis difficult in these species^16,17^.

Transfer of neural information over the corpus callosum, and other commissures, results in interhemispheric conduction delay^13^. For example, it has been estimated that an average-size myelinated fibre connecting the temporal lobes of the human brain has a conduction delay of more than 25 ms^13^. Higher length of a fibre tract connecting the two hemispheres results in a longer interhemispheric conduction delay compared to a fibre tract with shorter length. Therefore, Ringo suggested that time-sensitive processes that require a fast reaction to environmental demands underlie an evolutionary pressure to be controlled for by unilateral neural networks, as these minimize the time-consuming transfer of information to the contralateral side. This evolutionary pressure to conduct time-sensitive processes in one hemisphere then leads to the emergence of hemispheric asymmetry.

Importantly, most studies based on the Ringo hypothesis focussed on research in human subjects^18,19^. However, one of the key predictions of the hypothesis is decidedly comparative. Specifically, it is implied that the evolutionary pressure to develop hemispheric asymmetries is not the same for all Mammalian species but varies, amongst other factors, as a function of brain size. If this hypothesis is correct, Mammalian species with larger brains should exhibit more pronounced functional asymmetries. This should be the case since the interhemispheric conduction delay is directly dependent on the length of the fibre tracts that connect the two hemispheres, as long as fibre tract diameter size and myelinisation are invariable. Since the length of fibres connecting the two hemispheres increases when a brain has a larger overall size, larger brains should show more functional hemispheric asymmetries.

The aim of the present study was to empirically test this assumption. To this end, we have conducted a preregistered cross-species meta-regression analysis of limb preferences as a main form of functional hemispheric asymmetries^8–10^, with adult brain mass in g as predictor. The crucial factor that largely determines interhemispheric conduction delay is the length of the interconnecting fibres. Larger brain size or volume necessarily leads to longer interconnecting fibres. Thus, volumetric data would have been optimal but unfortunately are not widely available for many species. Data on adult brain mass is more widely available for many species and can serve, due to the direct correlation of brain mass and volume, as a suitable proxy for brain size. Since total brain neuron numbers have become available for many mammalian species over the recent years, we conducted additional analyses to check for a possible influence of neuron numbers. The aim of these analyses was to determine whether interhemispheric conduction delay has a major influence on the emergence of functional hemispheric asymmetries across mammals. Following the hypothesis by Ringo^13^, we assumed that the larger the average brain size of a species (and thus, the higher the brain mass), the more hemispheric asymmetries that species shows on average. We performed three meta-regressions with adult brain mass (in g) as a proxy for brain size as predictor, one for ambilateral vs. lateralized individuals, one for left-lateralized vs. non-left-lateralized individuals, and one for right-lateralized vs. non-right-lateralized. This results in the following three hypotheses:

1. Adult brain mass is a predictor for asymmetry in the meta-regression for ambilaterality. The directionality of the effect is negative, e.g., higher brain mass being related to fewer ambilateral individuals.
2. Adult brain mass is a predictor for asymmetry in the meta-regression for leftward lateralization. The directionality of the effect is positive, e.g., higher brain mass being related to more left-lateralized individuals.
3. Adult brain mass is a predictor for asymmetry in the meta-regression for rightward lateralization. The directionality of the effect is positive, e.g., higher brain mass being related to more right-lateralized individuals.

Following the same logic, we assume that species with a higher number of neurons in the brain should show more hemispheric asymmetries. This results in the following hypotheses:

1. Neuron number is a predictor for asymmetry in the meta-regression for ambilaterality. The directionality of the effect is negative, e.g., higher neuron number being related to less ambilateral individuals.
2. Neuron number is a predictor for asymmetry in the meta-regression for leftward lateralization. The directionality of the effect is positive, e.g., higher neuron number being related to more left-lateralized individuals.
3. Neuron number is a predictor for asymmetry in the meta-regression for rightward lateralization. The directionality of the effect is positive, e.g., higher neuron number being related to more right-lateralized individuals.

## Results and discussions

Overall, six meta-regressions were calculated (ambilaterality, leftward lateralization, and rightward lateralization, each with adult brain mass in g and neuron number as predictors). Data were collected from 28 different species (Table 1). Brain size data were available for all 28 species, neuron number data for only 17 species. Brain size and neuron number were significantly correlated (*r* = 0.989, *p* < 0.01).

**Table 1:** Studies on hemispheric asymmetries, brain size, and neuron numbers that were included in the meta-regressions.

Species	Study asymmetry	Study brain size	Study neuron number
Domestic sheep (*Ovis aries*)	^38^	^33^	–
Domestic pig (*Sus scrofa domesticus*)	^39^	^33^	^33^
Domestic dog (*Canis familiaris*)	^10^	^33^	^33^
Domestic cat (*Felis catus*)	^10^	^33^	^33^
Red Kangaroo (*Macropus rufus*)	^40^	^33^	–
Eastern Grey Kangaroo (*Macropus giganteus*)	^40^	^41^	–
Red-necked Wallaby (*Notamacropus rufogriseus*)	^42^	^43^	^43^
Goodfellow’s tree-kangaroo (*Dendrolagus goodfellowi*)	^40^	^43^	^43^
Common Marmoset (*Callithrix jacchus*)	^44^	^33^	^33^
Gorilla (*Gorilla gorilla*)	^45^	^33^	^46^
Orang utan (*Pongo pygmaeus*)	^45^	^33^	^46^
Chimpanzee (*Pan troglodytes*)	^45^	^33^	–
Bonobo (*Pan paniscus*)	^45^	^33^	–
Ring-tailed Lemur (*Lemur catta*)	^47^	^33^	–
Long-tailed Macaque (*Macaca fascicularis*)	^48^	^33^	^33^
Rhesus macaque (*Macaca mulatta*)	^48^	^41^	^49^
Southern pig-tailed macaque (*Macaca nemestrina*)	^48^	^50^	–
Eastern Grey Squirrel (*Sciurus carolinensis*)	^51^	^33^	^33^
Sugar glider (*Petaurus breviceps*)	^52^	^53^	–
Grey short-tailed opossum (*Monodelphis domestica*)	^52^	^53^	–
Squirrel Monkey (*Saimiri sciureus*)	^54^	^49^	^49^
Guinea baboon (*Papio papio*)	^55^	^41^	–
Olive baboon (*Papio anubis*)	^56^	^57^	–
Grey Mouse Lemur (*Microcebus murinus*)	^58^	^49^	^49^
Tufted Capuchin (*Cebus apella*)	^59^	^49^	^49^
House Mouse (*Mus musculus*)	^9^	^33^	^33^
Rat (*Rattus norvegicus*)	^9^	^33^	^33^
Human (*Homo sapiens*)	^8^	^33^	^33^

Numbers indicate the relevant references in the reference list.

**Fig. 1:**
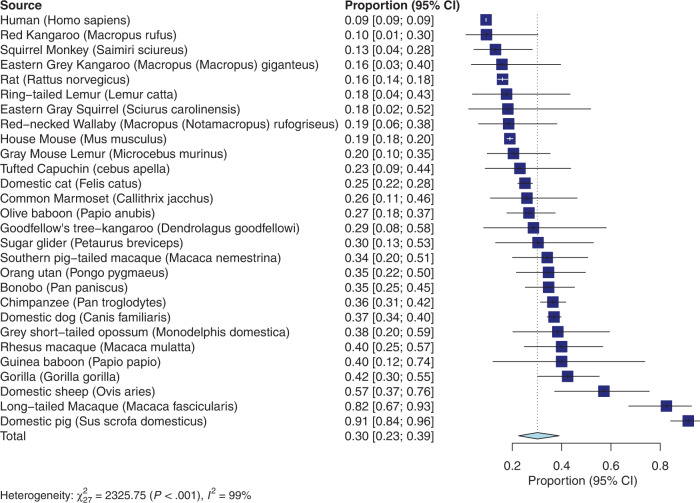
Results of the ambilaterality meta-analysis. Forest plot for the ambilaterality meta-analysis. Error bars show the 95% confidence interval.

For limb preferences and adult brain mass, the ambilaterality meta-analysis (see Fig. 1 for forest plot) revealed an overall proportion of ambilateral limb preferences across species that was 0.30 (95% confidence interval: 0.22–0.39). Thus, across species 30% of animals show an ambilateral preference. Significant heterogeneity across studies was detected (*Q*_(27)_ = 2325.75; *p* < 0.001). The meta-regression with brain mass as predictor did not reach significance (*F*_(1,26)_ = 0.4122; *p* = 0.53). This suggests that brain mass is not associated with the number of ambilateral individuals in a species.

**Fig. 2:**
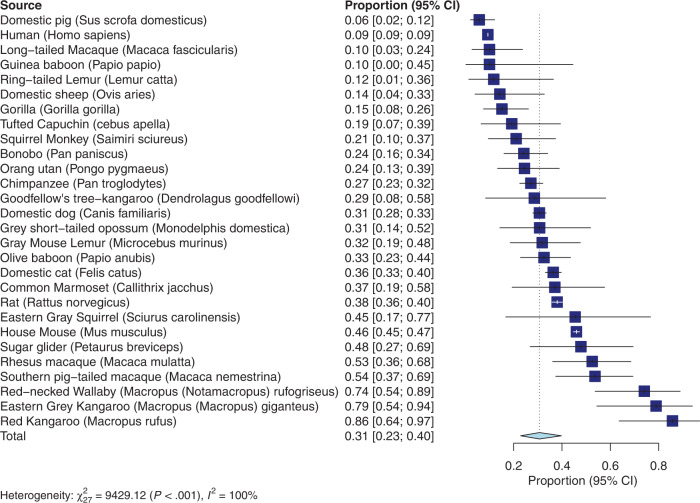
Results of the leftward lateralization meta-analysis. Forest plot for the leftward lateralization meta-analysis. Error bars show the 95% confidence interval.

The meta-analysis for leftward lateralization (see Fig. 2 for forest plot) revealed an overall proportion of leftward limb preferences across species that was 0.31 (95% confidence interval: 0.23–0.40). Thus, across species 31% of animals show a leftward preference. Significant heterogeneity across studies was detected (*Q*_(27)_ = 9429.12; *p* < 0.001). The meta-regression with brain mass as predictor reached significance (*F*_(1,26)_ = 6.77; *p* < 0.05), with a negative *t*-value of *t* = –2.60 for the predictor brain mass. This suggests that higher brain mass is associated with a lower number of individuals with a leftward preference in a species.

**Fig. 3:**
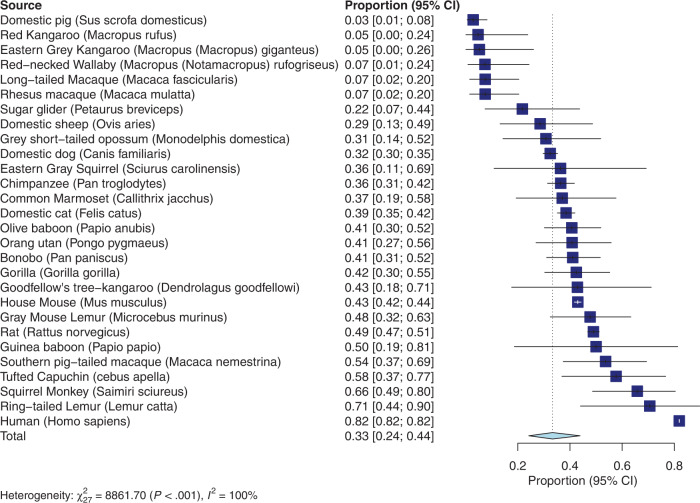
Results of the rightward lateralization meta-analysis. Forest plot for the rightward lateralization meta-analysis. Error bars show the 95% confidence interval.

The meta-analysis for rightward lateralization (see Fig. 3 for forest plot) revealed an overall proportion of rightward limb preferences across species that was 0.33 (95% confidence interval: 0.24–0.44). Thus, across species 33% of animals show a rightward preference. Significant heterogeneity across studies was detected (*Q*_(27)_ = 8861.70; *p* < 0.001). The meta-regression with brain mass as predictor reached significance (*F*_(1,26)_ = 4.42; *p* < 0.05), with a positive *t*-value of *t* = 2.10 for the predictor brain mass. This suggests that higher brain mass is associated with a higher number of individuals with a rightward preference in a species.

For the meta-analyses with neuron number as predictor in the meta-regression we do not present the forest plots, as this information is already included in the Figs. 1–3. The ambilaterality meta-analysis revealed an overall proportion of ambilateral limb preferences across species that was 0.30 (95% confidence interval: 0.19–0.44). Thus, across species 30% of animals showed an ambilateral preference. Significant heterogeneity across studies was detected (*Q*_(16)_ = 1900.33; *p* < 0.001). The meta-regression did not reach significance (*F*_(1,15)_ = 0.96; *p* = 0.34). This suggests that the number of neurons in the brain is not associated with the number of ambilateral individuals in a species.

The meta-analysis for leftward lateralization revealed an overall proportion of rightward limb preferences across species that was 0.28 (95% confidence interval: 0.19–0.38). Thus, across species 28% of animals showed a leftward preference. Significant heterogeneity across studies was detected (*Q*_(16)_ = 9091.78; *p* < 0.001). The meta-regression reached significance (*F*_(1,15)_ = 5.07; *p* < 0.05), with a negative *t*-value of *t* = −2.25 for the predictor number of neurons in the brain. This suggests that a higher number of neurons in the brain is associated with a lower number of individuals with a leftward preference in a species.

The meta-analysis for rightward lateralization revealed an overall proportion of rightward limb preferences across species that was 0.34 (95% confidence interval: 0.21–0.49). Thus, across species 34% of animals show a rightward preference. Significant heterogeneity across studies was detected (*Q*_(16)_ = 8216.07; *p* < 0.001). The meta-regression reached significance (*F*_(1,15)_ = 4.69; *p* < 0.05), with a positive *t*-value of *t* = 2.17 for the predictor number of neurons in the brain. This suggests that a higher number of neurons in the brain is associated with a higher number of individuals with a rightward preference in a species.

Taken together, the results for adult brain mass and neuron number in the brain paralleled each other completely, which is unsurprising given how high the correlation coefficient between them was. Thus, at least on the phylogenetic scale analyzed in the present study, the effect of the number of neurons cannot be separated from that of adult brain mass as an indicator of brain size. Thus, we will discuss the results of the two sets of meta-regressions together as they are largely identical.

For both variables, the predictor failed to show an association with ambilaterality but showed a significant positive association with the prevalence of rightward preferences in a species and a significant negative association with the prevalence of leftward preferences in a species. This finding is only partly in line with the Ringo hypothesis^13^ and the preregistered hypotheses of the present study. Two of the six preregistered hypotheses were confirmed. Brain mass and neuron number were statistically significant predictors for the number of animals with rightward lateralization and the directionality of the effect was positive (e.g., species with larger brains showed more rightward lateralization). The statistical tests for the hypotheses for ambilaterality, however, did not reach significance. For leftward lateralization we found effects that were significant but opposite to what was predicted in the hypotheses. This suggests that interhemispheric conduction delay may play a role in the evolution of functional hemispheric asymmetry but may not be as central as suggested by the Ringo hypothesis^13^.

The Ringo hypothesis predicts a general shift away from ambilaterality toward laterality in larger-brained species but makes no prediction on the direction of laterality. In contrast to this prediction, no significant ambilaterality effect was observed, suggesting that the evolution of asymmetry irrespective of its direction is not affected by brain size as suggested by the Ringo hypothesis^13^. The findings suggested a specific shift toward rightward limb preferences in larger-brained species and a reduced number of leftward limb preferences. This is most evident in humans with their distinct 90:10 distribution for right-handedness and left-handedness^8^. While the methodology of the present meta-regression study does not allow for causal inferences, it is evident that other factors than interhemispheric conduction delay need to be considered in the context of the evolution of hemispheric asymmetries. It could be speculated that sociality may be a factor that also plays a role, as it has been implied in both the evolution of brain size^20^ and the evolution of hemispheric asymmetries^21,22^. One leading theoretical account for the evolution of population-level hemispheric asymmetries within a species suggests that population-level asymmetries emerge as an evolutionarily stable strategy when organisms need to coordinate their behaviour with other asymmetrically behaving individuals^23,24^. In that context, the inter-individual interactions can generate evolutionarily stable strategies of lateralization at the individual- or population-level, depending on ecological contexts^25^. This implies that in particular social species should show population-level asymmetries towards one side, an idea that is supported by empirical evidence in both insects^21^ and fish^26^. In one study it was shown that the social honeybee shows hemispheric asymmetries on the behavioural and electrophysiological level, while the non-social mason bee does not. Moreover, the results of a study on handedness and learning how to fold asymmetric origami figures in humans supported the idea that matching hand preferences in the majority of the population evolved due to social learning processes^27^.

Importantly, in birds (which were not included in the present analysis), a recent study reported that Psittacine species with stronger left-foot preferences also have larger brains^28^. Interestingly, there is some evidence that in *Psittaciformes*, leftward foot preferences are more common than rightward foot preferences^29,30^. This suggests that increased brain size may lead towards a need for coherent lateralization on the side that is the dominant one in most individuals within a species. This implies that no specific evolutionary pressure to either converge to the left or the right side exists.

Several methodological aspects should be considered when interpreting the present results. Importantly, we did not have an equal distribution of animal species over different Mammalian orders, but primate species were clearly over-represented. This was due to data availability but could be problematic since primates tend to have larger brains than most other mammals. Also, there were several marsupial species included in the present study which are anatomically distinct from placental mammals as they do not have a corpus callosum. While the anterior commissure has a similar function to the corpus callosum in these species and the principal assumptions of the Ringo hypothesis are the same for all Mammalian orders, this anatomical difference may have affected data patterns. Moreover, other factors than brain size may have affected results, for example, gyrification, or neuron density. In addition, forelimb asymmetries are only one form of hemispheric asymmetries and many more have been investigated. By using this phenotype for the present meta-regression, many species with pronounced asymmetries in nervous systems structure and behaviour such as *C. elegans*^31^ that have no forelimbs were excluded from the analysis. Thus, it would be meaningful to also investigate other forms of hemispheric asymmetries in cross-species meta-regression.

## Materials and methods

### Preregistration

Prior to data collection, the study and the hypotheses were preregistered on the Open Science Framework Registries (URL: https://osf.io/ur52c) on April 7, 2022 under the title: “The Ringo model revisited: Using cross-species meta-regression to test the hypothesis that functional hemispheric asymmetries arise from interhemispheric conduction delay”. We later shortened the title to meet journal requirements. This did not change the content of the study. We have also slightly changed the wording of the hypotheses without changing their meaning following feedback on the preprint and feedback on the initial submission of the manuscript.

### Identification of relevant studies

Published literature was screened for eligible publications. For limb preferences this included a systematic analysis of limb preferences in non-human vertebrates published in 2013^32^ as well as the largest published meta-analysis of handedness in humans^8^. For neuron number and brain size this included a recent publication on brain size and neuron number in relation to yawn duration^33^. Additional papers on limb preferences and brain size / neuron numbers were identified using the scientific databases PubMed (https://www.ncbi.nlm.nih.gov/pubmed/) and ScienceDirect (https://www.sciencedirect.com/). All species for which either limb preferences or brain size / neuron number were available were screened for the other missing parameter. Search terms for limb preferences were “Handedness” OR “Pawedness” OR “Limb preferences” OR “asymmetry” AND species name. Search terms for brain size / neuron number were “Brain size” OR “Neuron number” AND species name. A species was included in the analyses when the following preregistered inclusion and exclusion criteria were met:

- A species was only included if data on limb preferences has been published. If more than one study on limb preferences in a species had been published, the meta-analysis with the largest n was used as reference. If no meta-analysis was available, the empirical study with the largest n was used as reference.
- In addition, data on adult brain mass in g as a proxy for brain size or neuron number needed to be available from openly available scientific resources (one of these two parameters was sufficient). If more than one study on brain mass / neuron number in a species had been published, the meta-analysis with the largest n was used as reference. If no meta-analysis was available, the empirical study with the largest n was used as reference.

During collection of relevant studies, we realized that in some situations, the preregistered exclusion and inclusion criteria were not specific enough. We therefore also applied the following set of rules:

- Minimum sample size: For asymmetry data, studies with a sample size *n* < 10 were excluded from the analysis as they were deemed too unreliable.
- Asymmetry measure: The most widely used method to assess limb preferences in animals is the food reaching task^34^. If more than one asymmetry measure was used in a specific study in a specific species, data from unimanual food reaching or the task most similar to food reaching was chosen. If two or more different studies with similar sample size were published on limb preferences in a species, and the studies had different experimental paradigms to assess limb preferences, the study that used food reaching was used for further analysis.
- Age: Only studies testing adult animals were included in the analysis to avoid development effects.
- Classification system: If a study included an analysis that compared between left- and right-pawed individuals and a second analysis with three categories (left-pawed, right-pawed, and ambidextrous), only the latter was used for this paper.
- Study language: Only studies written in English and German were considered.

**Fig. 4:**
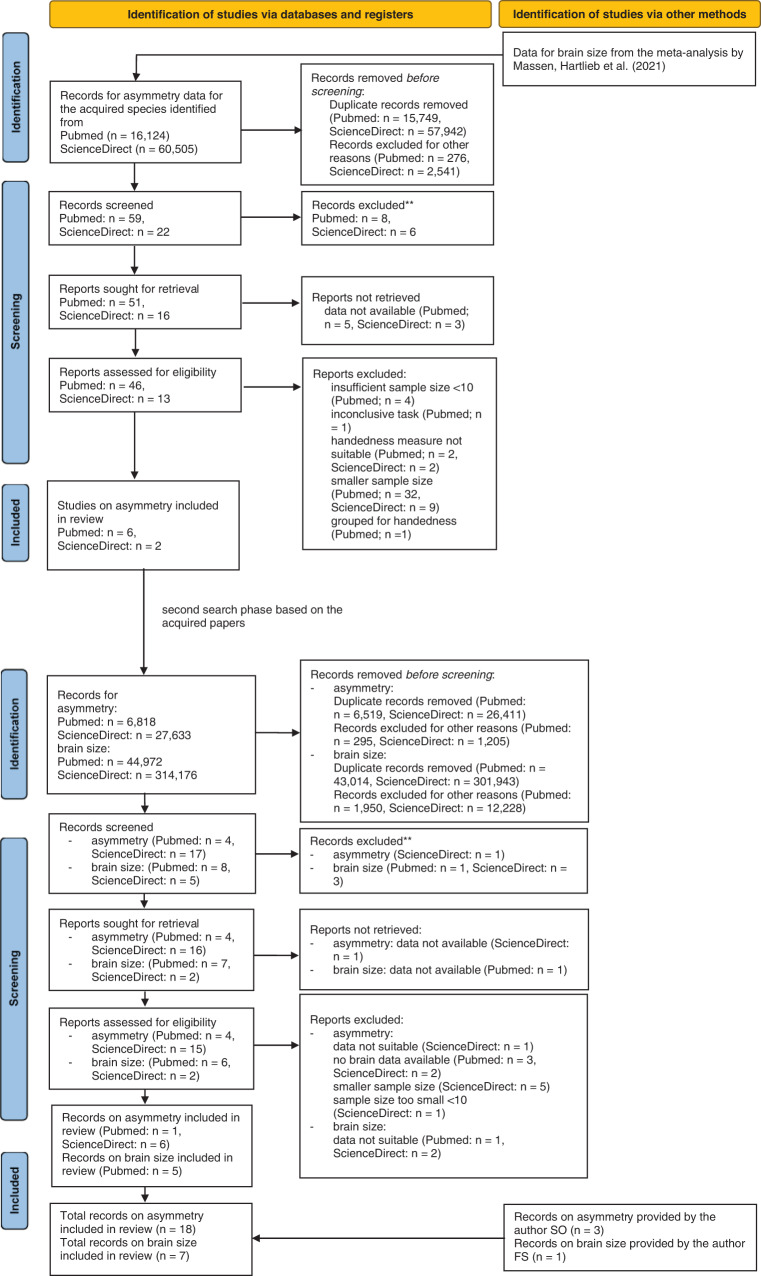
Study identification process for the brain size analyses. Flow chart for the study identification process via databases and registers for brain mass as a proxy for brain size. Note that the numbers reported here are lower than the overall number of studies in the meta-regressions since many studies were identified from review articles.

**Fig. 5:**
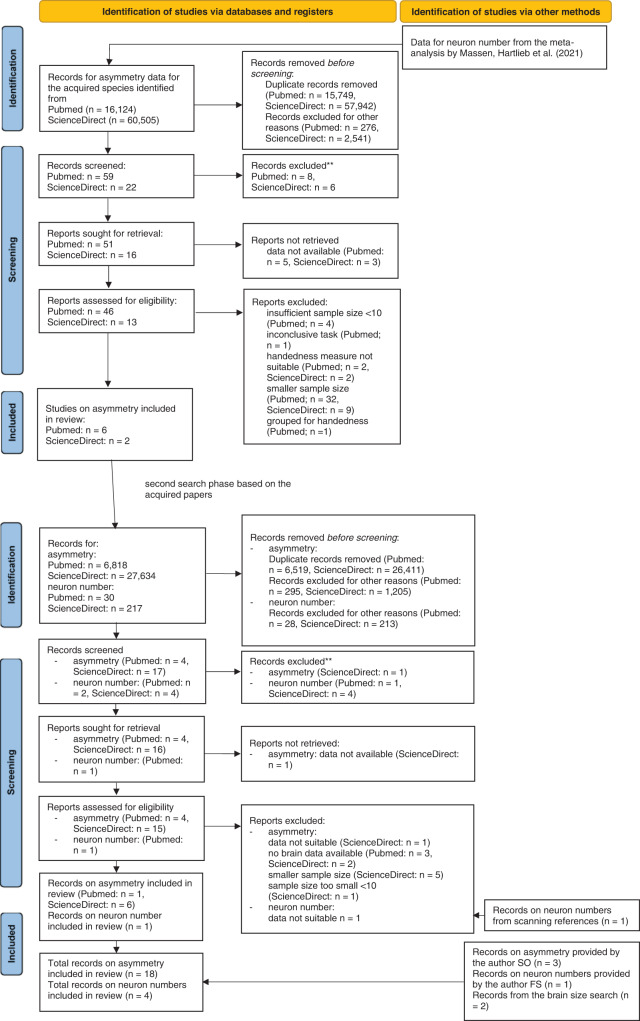
Study identification process for the neuron number analyses. Flow chart for the study identification process via databases and registers for neuron number. Note that the numbers reported here are lower than the overall number of studies in the meta-regressions since many studies were identified from review articles.

In a second step, the acquired papers were scanned for further animal species in which asymmetry data or brain size/neuron numbers were available. The name of the species was then searched in combination with “brain size” OR “neuron number” or the asymmetry search terms used before. One study on brain size and neuron numbers was provided by the author FS based on prior knowledge^35^. Figure 4 shows the flow chart for the identification of studies the limb preferences and brain size meta-regressions and Fig. 5 shows the flow chart for the limb preferences and neuron number meta-regressions. Flow charts were based upon the PRISMA statement flow chart^36^ but were customized to fit the specific data collection procedure with two different phenotypes in the present study.

### Meta-regressions

Overall, we conducted 6 different preregistered random-effects meta-analyses following standard protocol^37^ in R (https://www.r-project.org/) and R Studio (https://www.rstudio.com/). In each meta-analysis we performed meta-regression, either with brain size or with neuron numbers. This resulted in the following 6 analyses:

1. Random-effects meta-analysis of ambilaterality across species with adult brain mass as proxy for brain size as predictor in meta-regression
2. Random-effects meta-analysis of for leftward lateralization species with adult brain mass as proxy for brain size as predictor in meta-regression
3. Random-effects meta-analysis of for rightward lateralization across species with adult brain mass as proxy for brain size as predictor in meta-regression
4. Random-effects meta-analysis of ambilaterality across species with neuron number as predictor in meta-regression
5. Random-effects meta-analysis of for leftward lateralization across species with neuron number as predictor in meta-regression
6. Random-effects meta-analysis of for rightward lateralization individuals across species with neuron number as predictor in meta-regression

The standard *p* < 0.05 criteria for determining significance was used in meta-analysis and meta-regression.

### Reporting summary

Further information on research design is available in the Nature Portfolio Reporting Summary linked to this article.

## Supplementary information

Ocklenburg_Peer Review File

Reporting Summary

## Data Availability

No new experimental data were collected for this meta-analysis. All data gathered from previously published manuscripts, as well as the R code used to calculate the meta-analyses and meta-regressions are freely available on the OSF page of this project (https://osf.io/kq596/).
