# Supplementary material for: Hemispheric asymmetries and brain size in mammals
Source: Commun Biol. 2023 May 15;6:521. doi: 10.1038/s42003-023-04894-z (PMC10185570; doi:10.1038/s42003-023-04894-z)
Supplement: Supplementary file 1 — Ocklenburg_Peer Review File [file 42003_2023_4894_MOESM1_ESM.pdf]

nature portfolio

Peer Review File

Hemispheric asymmetries and brain size in mammals: A cross-species meta-regression

Open Access This file is licensed under a Creative Commons Attribution 4.0 International License, which permits use, sharing, adaptation, distribution and reproduction in any medium or format, as long as you give appropriate credit to the original author(s) and the source, provide a link to the Creative Commons license, and indicate if changes were made. In the cases where the authors are anonymous, such as is the case for the reports of anonymous peer reviewers, author attribution should be to 'Anonymous Referee' followed by a clear attribution to the source work. The images or other third party material in this file are included in the article's Creative Commons license, unless indicated otherwise in a credit line to the material. If material is not included in the article's Creative Commons license and your intended use is not permitted by statutory regulation or exceeds the permitted use, you will need to obtain permission directly from the copyright holder. To view a copy of this license, visit http://creativecommons.org/licenses/by/4.0/.

Reviewers' comments:

Reviewer #1 (Remarks to the Author):

I think this paper is interesting, though perhaps the fact that Ringo’s hypothesis is untenable as an explanation for the evolution of brain asymmetry is stated quite shyly, given the authors’ results. There are also some general issues in the paper that deserves some discussion/clarification.

Line 52. Actually, evidence on cats and dogs show clear directional asymmetries but in different directions in males and females (of course, when lumped together any directional bias disappears). Also, using pawedness as the unique measure for brain asymmetry seems to me misleading. Most of the evidence for asymmetries in animal species are observed for different behaviors or as direct measurements in the nervous system. Thus, using asymmetries in the use of forelimbs as the only evidence for asymmetries in the nervous system seems to me questionable: C. elegans have been proved to have brain asymmetries at the population level and the same is true in vertebrates such as zebrafish, and these are among the most promising animal models for the investigation of neurobiology and genetics of brain asymmetries. Clearly, we cannot expect to use handedness as a proper behavioural measure in these species.

Line 61-62: I am puzzled by these arguments for birds do not have any equivalent of a callosum and interhemispheric connections is not occurring for homologous brain regions in the pallium as occurs in mammals. In spite of this they have striking directional (population-level) asymmetries. Similar arguments run for fish and reptiles (and even more so for invertebrates, see also above).

Line 78: it seems to me that much more than brain size is the presence or absence of a callosum that would provide a proper test of the theory: How can this theory be maintained for species without callosum and/or with little inter-hemispheric communication such as fish or reptiles?

Line 86: Again, limb asymmetries are used as a proxy for brain asymmetry. But this is untenable for a variety of species proved to be brain lateralized.

Line 202-204: It seems clear to me that the lack of association with ambilaterality is the crucial finding. It suggests that the evolution of asymmetry as such (irrespective of its direction) is not affected by the variables hypothesized by Ringo. Indeed, as stressed by the authors “The Ringo hypothesis predicts a general shift away from ambilaterality toward laterality in larger-brained species but makes no prediction on the direction of laterality.”

Linee 218: I think the first original paper of the theory is however Ghirlanda, S., Vallortigara, G. (2004). The evolution of brain lateralization: A game theoretical analysis of population structure. Proceedings of the Royal Society B, 271: 853-857. The one the authors quoted is a successive extension of it to competitive/cooperative interactions

Line 220: Actually, there is evidence also for vertebrates, i.e. correlation between shoaling in fish with their brain asymmetry, e.g. Bisazza et al. (2000). Population lateralization and social behaviour: A study with sixteen species of fish. Laterality, 3: 269-284.

Line 220: “This implies that in particular social species should show population level asymmetries towards one side,…”. Actually, the theory is a bit more sophisticated than that. The idea is that the nature of inter-individuals interaction can generate evolutionarily stable strategies of lateralization at

the individual- or population-level, depending
on ecological contexts; in this regards individual-level and population-level lateralization should be considered as two aspects of the same continuum in the game. See e.g. Frasnelli, E., Vallortigara, G. (2018). Individual-level and population-level lateralization: Two sides of the same
coin. Symmetry, 10, 739.

Reviewer #2 (Remarks to the Author):

This paper reports an interesting study examining the relationship between brain mass, neurone number and limb preference in a range of species. It finds that rightward limb preference correlates positively with brain mass, and neurone number, and leftwards limb preference correlates negatively with these same factors.
Although the findings could be compromised by the amount of data, and number of species, used in the analyses, this study is a worthwhile first report of this relationship in mammalian species. It complements a previous study which found an association between brain size and strength of foot preference in avian species. Moreover, the authors point out the limitations of their study.

'52-55: This is correct for the species mentioned but ambilaterality is relatively rare in, for example, adult common marmosets, and this would be worth mentioning (e.g. Hook and Rogers, 2008, J comp. Psychol. 122, 41-51). Age also plays a role in this: ambilaterality is more common in young marmosets.

106: Correct 'less' to 'fewer'.

145-147: I think left and right must be confused in these sentences. Probably, 'rightward' in line 145 should be 'leftward'.

Reviewer #3 (Remarks to the Author):

Thanks for inviting me to review the manuscript entitled " Hemispheric asymmetries and brain size: A cross-species meta-regression" This is an interesting study that tests the Ringo hypothesis for the origin of brain lateralization in a comparative framework. The study analyses data obtained from previous studies following rigorous and well explained criteria. The topic is relevant given the controversies around the evolutionary origin of brain asymmetries. However, I have some concerns with part of the analyses performed that need to be addressed. I hope my comments help to improve the manuscript.

The rationale for comparing brain volume and asymmetry is well explained and based on Ringo´s hypothesis. However, incorporating the number of neurons as an explanatory variable is less convincing. The authors only mention in the introduction that "Since total brain neuron numbers have become available for many mammalian species over the recent years, we conducted additional analyses, to check for a possible influence of neuron numbers". Please, clarify the results expected for this factor. The authors mention that they follow the same logic, but it is not clear why you expect that "species with a higher number of neurons in the brain should show more hemispheric asymmetries." I would expect that as the number of neurons increases, the white matter, and thus

the connectivity, also increases.

Another variable that can be of greater interest to the problem under study is neuron density, given that brains of similar size can differ in neuron number.

I also wonder whether brain volume and neuron number are associated. So, I recommend checking for that association because the results obtained are similar using either variable, which could be the result of both of them mainly representing changes in brain volume.

It is unclear why the authors calculate simple linear regressions for volume and neuron number separately instead of performing a multivariate regression. I recommend repeating the analyses of neuron number controlling for brain size.

Given that one of the main conclusions is that the results for adult brain mass and neuron number paralleled each other, the discussion might be need to be revised according to the results obtained.

Minor comments:
The phrase “preregistered hypotheses” is used in the manuscript, but I´m not sure whether the word preregistered is the most adequate to refer to the hypothesis proposed in the study.

I also wonder whether brain volume and neuron number are associated with the sample analyzed. I recommend checking for that association. The results obtained are similar using either variable, which could be the result of both of them mainly representing changes in brain volume.

<u>Rebuttal letter</u>

<u>Reviewers' comments:</u>

<u>Reviewer #1</u>

Reviewer's suggestion #01:
I think this paper is interesting, though perhaps the fact that Ringo's hypothesis is untenable as an explanation for the evolution of brain asymmetry is stated quite shyly, given the authors' results. There are also some general issues in the paper that deserves some discussion/clarification.

Authors´ response #01:
We thank the reviewer for the generally positive evaluation of our manuscript and the helpful comments that improved our manuscript significantly.

Reviewer's suggestion #02:
Line 52. Actually, evidence on cats and dogs show clear directional asymmetries but in different directions in males and females (of course, when lumped together any directional bias disappears). Also, using pawedness as the unique measure for brain asymmetry seems to me misleading. Most of the evidence for asymmetries in animal species are observed for different behaviors or as direct measurements in the nervous system. Thus, using asymmetries in the use of forelimbs as the only evidence for asymmetries in the nervous system seems to me questionable: C. elegans have been proved to have brain asymmetries at the population level and the same is true in vertebrates such as zebrafish, and these are among the most promising animal models for the investigation of neurobiology and genetics of brain asymmetries. Clearly, we cannot expect to use handedness as a proper behavioural measure in these species.

Authors´ response #02:
We agree with the reviewer that there is a wide variety of hemispheric asymmetries across all kinds of species, even in those that do not have forelimbs like *C. Elegans*.
However, the reason we choose forelimb asymmetries is more methodological than conceptual. In order to conduct a meta-analysis, we need a phenotype that is similar across species and mathematically scales in the same way. This is why we chose forelimb asymmetries, simply because it was the phenotype with the most comparable datapoints across species. We cannot integrate C. Elegans olfactory neuron asymmetry, zebrafish anatomical asymmetries, and so on in this model, because the dependent variables are different and scale differently. These would, however, be interesting models for future meta-analyses with cross-species meta-regression. We, therefore, integrated a new section in the discussion outlining the reviewer's ideas.

It reads:
"In addition, forelimb asymmetries are only one form of hemispheric asymmetries and many more have been investigated. By using this phenotype for the present meta-regression, many species with pronounced asymmetries in nervous systems structure and behavior such as C. elegans 26 that have no forelimbs were excluded from the analysis. Thus, it would be meaningful to also investigate other forms of hemispheric asymmetries in cross-species meta-regression."

Reviewer's suggestion #03:
Line 61-62: I am puzzled by these arguments for birds do not have any equivalent of a callosum and interhemispheric connections is not occurring for homologous brain regions in

the pallium as occurs in mammals. In spite of this they have striking directional (population-level) asymmetries. Similar arguments run for fish and reptiles (and even more so for invertebrates, see also above).

Authors´ response #03:
The original Ringo et al paper from 1990 is focused on human brain asymmetries and there is no mention to what extent that theory can be extended to non-Mammalian species, with different commissural systems. Given that the theory is centered around projections over the corpus callosum not present in non-mammalian vertebrates and, based on the currently available data, is also not substituted by other commissures (e.g., the anterior commissure in metatherians – Suarez et al. 2014, 2018), it would be difficult to apply the Ringo theory in these species. Furthermore, we do not claim at all that the Ringo theory alone can explain strength and the emergence of hemispheric asymmetries. It rather describes one of many potential factors playing a role in asymmetry formation and extent. As the reviewer has mentioned, there are several striking examples for hemispheric asymmetries outside the mammalian clade, including, for example, highly persistent eye and claw lateralization in pigeons and parrots, respectively. While these could be considered under the umbrella of the Ringo hypothesis as extreme examples for very little interhemispheric connectivity, it is questionable how the remaining connectivity over the other pallial commissures (hippocampal and anterior) scales with brain size or neuron number and how this relates to mammalian data. We therefore refrained from adding non-mammalian species to the analysis but plan to do separate analyses, for example, in avian species as soon as there is enough data available.

We deleted the argument related to birds in the introduction and now state:

“Since the corpus callosum evolved in placental mammals, the more basal mammalian Metatheria do not possess a corpus callosum yet. Instead, the anterior commissure constitutes the major interhemispheric pathway [15] and shows a connection pattern similar to the corpus callosum in placental mammals [16]. While other vertebrate species like birds, reptiles, amphibians, and fish do have similar commissural systems compared to Metatherians, they differ in extent and projection pattern, rendering the application of the Ringo hypothesis difficult in these species.”

We still are convinced that it is worthwhile to test this theory empirically given its major importance in the literature on human hemispheric asymmetries.

Reviewer’s suggestion #04:
Line 78: it seems to me that much more than brain size is the presence or absence of a callosum that would provide a proper test of the theory: How can this theory be maintained for species without callosum and/or with little inter-hemispheric communication such as fish or reptiles?

Authors´ response #04:
See response to #03

Reviewer’s suggestion #05:
Line 86: Again, limb asymmetries are used as a proxy for brain asymmetry. But this is untenable for a variety of species that proved to be brain lateralized.

Authors´ response #05:
As mentioned above, we have added a section discussing this issue to the Discussion section.

Reviewer's suggestion #06:
Line 202-204: It seems clear to me that the lack of association with ambilaterality is the crucial finding. It suggests that the evolution of asymmetry as such (irrespective of its direction) is not affected by the variables hypothesized by Ringo. Indeed, as stressed by the authors "The Ringo hypothesis predicts a general shift away from ambilaterality toward laterality in larger-brained species but makes no prediction on the direction of laterality."

Authors´ response #06:
We agree with the reviewer and have expanded this section.

It now reads:

"The Ringo hypothesis predicts a general shift away from ambilaterality toward laterality in larger-brained species but makes no prediction on the direction of laterality. In contrast to this prediction, no significant ambilaterality effect was observed, suggesting that the evolution of asymmetry irrespective of its direction is not affected by brain size as suggested by the Ringo hypothesis 13."

Reviewer's suggestion #07:
Line 218: I think the first original paper of the theory is however Ghirlanda, S., Vallortigara, G. (2004). The evolution of brain lateralization: A game theoretical analysis of population structure. Proceedings of the Royal Society B, 271: 853-857. The one the authors quoted is a successive extension of it to competitive/cooperative interactions

Authors´ response #07:
In accordance with the reviewer's suggestion, we have now added a reference to the Ghirlanda et al. (2004) paper in this section.

Reviewer's suggestion #08:
Line 220: Actually, there is evidence also for vertebrates, i.e. correlation between shoaling in fish with their brain asymmetry, e.g. Bisazza et al. (2000). Population lateralization and social behaviour: A study with sixteen species of fish. Laterality, 3: 269-284.

Authors´ response #08:
In accordance with the reviewer's suggestion, we have added this interesting study to this section.
The sentence now reads:
"This implies that social species in particular should show population-level asymmetries towards one side, an idea that is supported by empirical evidence in both insects [20] and fish [23]."
New reference:
23. Bisazza, A., Cantalupo, C., Capocchiano, M. & Vallortigara, G. Population lateralization and social behaviour: a study with 16 species of fish. Laterality 5, 269–284; 10.1080/713754381 (2000).

Reviewer's suggestion #09:
Line 220: "This implies that in particular social species should show population-level asymmetries towards one side,…".
Actually, the theory is a bit more sophisticated than that. The idea is that the nature of inter-individuals interaction can generate evolutionarily stable strategies of lateralization at the

individual- or population-level, depending on ecological contexts; in this regard, individual-level and population-level lateralization should be considered as two aspects of the same continuum in the game.
See e.g. Frasnelli, E., Vallortigara, G. (2018). Individual-level and population-level lateralization: Two sides of the same coin. Symmetry, 10, 739.

Authors´ response #09:
We have now added this information to the manuscript, including a citation of the mentioned paper.

The new section reads:

"In that context, the inter-individual interactions can generate evolutionarily stable strategies of lateralization at the individual- or population-level, depending on ecological contexts[24]*."*

New reference:

Frasnelli, E. & Vallortigara, G. Individual-Level and Population-Level Lateralization: Two Sides of the Same Coin. Symmetry 10, 739; 10.3390/sym10120739 (2018).

<u>Reviewer #2</u>

Reviewer's suggestion #01:
This paper reports an interesting study examining the relationship between brain mass, neuron number and limb preference in a range of species. It finds that rightward limb preference correlates positively with brain mass, and neuron number, and leftward limb preference correlates negatively with these same factors. Although the findings could be compromised by the amount of data, and the number of species, used in the analyses, this study is a worthwhile first report of this relationship in mammalian species. It complements a previous study that found an association between brain size and strength of foot preference in avian species. Moreover, the authors point out the limitations of their study.

Authors´ response #01:
We thank the reviewer for the generally positive evaluation of our manuscript and their helpful comments that improved our manuscript significantly.

Reviewer's suggestion #02:
'52-55: This is correct for the species mentioned but ambilaterality is relatively rare in, for example, adult common marmosets, and this would be worth mentioning (e.g. Hook and Rogers, 2008, J comp. Psychol. 122, 41-51). Age also plays a role in this: ambilaterality is more common in young marmosets.

Authors´ response #02:
In accordance with the reviewer's suggestion, we added this relevant finding to the text in the introduction in the mentioned section.
The new sentence reads:
"In common marmosets, ambilaterality is relatively rare and age-dependent, with younger animals showing ambilaterality more commonly in visuospatial reaching tasks [12]."
We referenced the mentioned paper:
12. Hook, M. A. & Rogers, L. J. Visuospatial reaching preferences of common marmosets (Callithrix jacchus): an assessment of individual biases across a variety of tasks. Journal of comparative psychology (Washington, D.C. : 1983) 122, 41–51; 10.1037/0735-7036.122.1.41 (2008).

Reviewer's suggestion #03:
106: Correct 'less' to 'fewer'.

Authors´ response #03:
Corrected in accordance with the reviewer's suggestion.

Reviewer's suggestion #04:
145-147: I think left and right must be confused in these sentences. Probably, 'rightward' in line 145 should be 'leftward'.

Authors´ response #04:
We are thankful that the reviewer noticed this mistake. Yes, "rightward" in line 145 indeed should be "leftward". We corrected this now.

<u>Reviewer #3</u>

Reviewer’s suggestion #01:
Thanks for inviting me to review the manuscript entitled “ Hemispheric asymmetries and brain size: A cross-species meta-regression“ This is an interesting study that tests the Ringo hypothesis for the origin of brain lateralization in a comparative framework. The study analyses data obtained from previous studies following rigorous and well explained criteria. The topic is relevant given the controversies around the evolutionary origin of brain asymmetries. However, I have some concerns with part of the analyses performed that need to be addressed. I hope my comments help to improve the manuscript.

Authors´ response #01:
We thank the reviewer for the generally positive evaluation of our manuscript and their helpful comments that improved our manuscript significantly.

Reviewer’s suggestion #02:
The rationale for comparing brain volume and asymmetry is well explained and based on Ringo´s hypothesis. However, incorporating the number of neurons as an explanatory variable is less convincing. The authors only mention in the introduction that “Since total brain neuron numbers have become available for many mammalian species over the recent years, we conducted additional analyses, to check for a possible influence of neuron numbers”. Please, clarify the results expected for this factor. The authors mention that they follow the same logic, but it is not clear why you expect that “species with a higher number of neurons in the brain should show more hemispheric asymmetries.” I would expect that as the number of neurons increases, the white matter, and thus the connectivity, also increases.

Authors´ response #02:
We agree with the reviewer that the theoretical basis for brain size is more convincing. Our main focus was clearly on brain size, as this is what the Ringo hypothesis is about. We did include neuron number since a growing number of papers have criticized brain size as being a rather imprecise measure in relation to brain function and since exactly this suitable data set was available, we thought it would be interesting to add more relevant information to the manuscript.

Reviewer’s suggestion #03:
Another variable that can be of greater interest to the problem under study is neuron density, given that brains of similar size can differ in neuron number.

Authors´ response #03:
We agree with the reviewer and have added this suggestion to the discussion. The sentence reads:

“Moreover, other factors than brain size may have affected results, for example, gyrification, or neuron density.”

Reviewer’s suggestion #04:
I also wonder whether brain volume and neuron number are associated. So, I recommend checking for that association because the results obtained are similar using either variable, which could be the result of both of them mainly representing changes in brain volume.

Authors´ response #04:
Since brain size was available for 28 species and neuron number only for 17, this analysis could only be performed for a subset of species (17). We found the correlation coefficient r to 0.989 and the p-value to be $p<0.001$.

We have added this information to the manuscript, the sentence reads:

"Brain size and neuron number were significantly correlated ($r=0.989$, $p<0.01$)."

This indicates that the reviewer is correct and that both variables largely cover the same variance.

Reviewer's suggestion #05:
It is unclear why the authors calculate simple linear regressions for volume and neuron number separately instead of performing a multivariate regression. I recommend repeating the analyses of neuron number controlling for brain size.

Authors´ response #05:
There may be a misunderstanding here. We did not perform simple linear regression on participant data. In the current study, we performed a meta-analysis on aggregated data for each species, e.g., data for humans were one data point containing the handedness percentage and the number of data points from the original study (about 2.3 million). Based on the meta-analyses, we performed meta-regression, a specific regression analysis specific for these meta-data. If we used traditional multivariate regression techniques, we would grossly underestimate the real n, making these methods invalid for this specific data. Unfortunately, there currently is no multivariate meta-regression available to our knowledge, which is why we needed to calculate two meta-regressions. Moreover, the Ringo hypothesis specifically states a relation with brain size, making it important to test this variable in isolation from a theoretical point of view.

Reviewer's suggestion #06:
Given that one of the main conclusions is that the results for adult brain mass and neuron number paralleled each other, the discussion might be need to be revised according to the results obtained.

Authors´ response #016:
See our previous answer for why this analysis could not be performed.

Minor comments:
Reviewer's suggestion #07:
The phrase "preregistered hypotheses" is used in the manuscript, but I´m not sure whether the word preregistered is the most adequate to refer to the hypothesis proposed in the study.

Authors´ response #07:
As stated in the section "Preregistration", we have preregistered the study and all 3 specific hypotheses tested in our data on the Open Science Framework Registries (URL: https://osf.io/ur52c) prior to data collection. Thus, the phrase "preregistered hypotheses" is correct.

Reviewer's suggestion #08:
I also wonder whether brain volume and neuron number are associated with the sample analyzed. I recommend checking for that association. The results obtained are similar using either variable, which could be the result of both of them mainly representing changes in brain volume.

Authors´ response #08:
Since brain size was available for 28 species and neuron number only for 17, this analysis could only be performed for a subset of species (17). We found the correlation coefficient r to 0.989 and the p-value to be $p<0.001$.

We have added this information to the manuscript, the sentence reads:

"Brain size and neuron number were significantly correlated ($r=0.989$, $p<0.01$)."

This indicates that the reviewer is correct and that both variables largely cover the same variance.

REVIEWERS' COMMENTS:

Reviewer #1 (Remarks to the Author):

I think the authors addressed adequately all my comments, and I am happy therefore to recommend this paper for publication as it stands.

Reviewer #3 (Remarks to the Author):

I thank the authors for taking into account my previous comments, which were either addressed in the new version of the manuscript or replied in the rebuttal letter. I still think the hypotheses about the association between the number of neurons and the functional lateralization are not well founded. The finding of a high association between this variable and brain volume suggests that, at least at the phylogenetic scale analyzed here, the effect of the number of neurons cannot be separated from brain size. Consequently, only three hypotheses with biological meaning are being tested. Given that organ size is associated with several phenotypic traits, it needs to be accounted for in comparative studies. I understand there are methodological limitations, but these aspects should be discussed in the manuscript. I have a few minor comments:

1. I think the percentages of left-handed and right-handed are swapped in the following sentence: “For example, humans show a clear population-level asymmetry for handedness, with roughly 90% of individuals being left-handed and 10% being right-handed..”

2. The title is too broad regarding the taxonomic level analyzed. Given that the study includes a rather reduced number of species representative of very diverse groups of mammals including some marsupials, it is important to make clear the extent of the conclusions attained. Probably adding a reference to mammals in the title or the key words could help to narrow the scope of the study.

Response To Reviews Revision 2

Reviewer #1:

Remark to the Author #1:

I think the authors addressed adequately all my comments, and I am happy therefore to recommend this paper for publication as it stands.

Reply by the authors #1:

We thank the reviewer for their positive evaluation of the manuscript.

Reviewer #3

General evaluation

I thank the authors for taking into account my previous comments, which were either addressed in the new version of the manuscript or replied in the rebuttal letter. I still think the hypotheses about the association between the number of neurons and the functional lateralization are not well founded. The finding of a high association between this variable and brain volume suggests that, at least at the phylogenetic scale analyzed here, the effect of the number of neurons cannot be separated from brain size. Consequently, only three hypotheses with biological meaning are being tested. Given that organ size is associated with several phenotypic traits, it needs to be accounted for in comparative studies. I understand there are methodological limitations, but these aspects should be discussed in the manuscript.

Reply by the authors #1:

We agree with the reviewer that due to the high correlation between neuron number and brain size there are only three biologically meaningful hypotheses tested.

However, the manuscript was preregistered on OSF with six hypotheses, and omitting half of them would not be in line with the methodological standards of preregistered studies. We only knew after calculating the correlation that there is a strong association between the two variables, not at the time of preregistration. Thus, we strongly prefer to keep the six hypotheses in the manuscript.

As we agree with the reviewer, we have added the following text to the discussion:

"Taken together, the results for adult brain mass and neuron number in the brain paralleled each other completely, which is unsurprising given how high the correlation coefficient between them was. Thus, at least on the phylogenetic scale analyzed in the present study, the effect of the number of neurons cannot be separated from that of adult brain mass as an indicator of brain size. Thus, we will discuss the results of the two sets of meta-regressions together as they are largely identical."

Remark to the Author #1:

I have a few minor comments:

1. I think the percentages of left-handed and right-handed are swapped in the following sentence: “For example, humans show a clear population-level asymmetry for handedness, with roughly 90% of individuals being left-handed and 10% being right-handed..”

Reply by the authors #1:

We thank the reviewer for informing us about this error. The sentence has been corrected and now reads:

“For example, humans show a clear population-level asymmetry for handedness, with roughly 90% of individuals being right-handed and 10% being left-handed.”

Remark to the Author #2:

2. The title is too broad regarding the taxonomic level analyzed. Given that the study includes a rather reduced number of species representative of very diverse groups of mammals including some marsupials, it is important to make clear the extent of the conclusions attained. Probably adding a reference to mammals in the title or the key words could help to narrow the scope of the study.

Reply by the authors #1:

We have added „in mammals“ to the title and the abstract and have also added “mammals” to the keywords.
